# Marital status, an independent predictor for survival of gastric neuroendocrine neoplasm patients: a SEER database analysis

**DOI:** 10.1186/s12902-020-00565-w

**Published:** 2020-07-23

**Authors:** Yu-Jie Zhou, Xiao-Fan Lu, Kenneth I. Zheng, Qi-Wen Wang, Jin-Nan Chen, Qing-Wei Zhang, Fang-Rong Yan, Xiao-Bo Li

**Affiliations:** 1 Division of Gastroenterology and Hepatology, Key Laboratory of Gastroenterology and Hepatology, Ministry of Health, Shanghai Institute of Digestive Disease, Renji Hospital, School of Medicine, Shanghai Jiao Tong University, 160 Pujian Road, Shanghai, 200127 China; 2 State Key Laboratory of Natural Medicines, Research Center of Biostatistics and Computational Pharmacy, China Pharmaceutical University, Nanjing, China; 3 Department of Hepatology, the First Affiliated Hospital of Wenzhou Medical University, Wenzhou, China

**Keywords:** Gastric neuroendocrine neoplasms, Marriage, Propensity score matching

## Abstract

**Background:**

Marital status proves to be an independent prognostic factor in a variety of cancers. However, its prognostic impact on gastric neuroendocrine neoplasms (G-NEN) has not been investigated.

**Methods:**

We identified 3947 G-NEN patients from the Surveillance, Epidemiology, and End Results (SEER) database. Meanwhile, propensity scores for marital status were used to match 506 unmarried patients with 506 married patients. We used Kaplan–Meier method and multivariate Cox regression to analyse the association between marital status and the overall survival (OS) and G-NEN cause-specific survival (CSS) before matching and after matching.

**Results:**

Married patients enjoyed better OS and CSS, compared with divorced/separated, single, and widowed patients. Multivariate Cox regression analysis indicated that unmarried status was associated with higher mortality hazards for both OS and CSS among G-NEN patients. Additionally, widowed individuals had the highest risks of overall (adjusted hazard ratio (HR): 1.56, 95% confidence interval (CI): 1.35–1.81, *P* < 0.001) and cancer-specific mortality (adjusted HR: 1.33, 95% CI: 1.05–1.68, *P* = 0.02) compared to other unmarried groups in both males and females. Furthermore, unmarried status remained an independent prognostic and risk factor for both OS (HR 1.51, 95% CI 1.19–1.90, *P* = 0.001) and CSS (HR 1.50, 95% CI 1.10–2.05, *P* = 0.01) in 1:1 propensity score-matched analysis.

**Conclusion:**

Marital status was an independent prognostic factor for G-NEN. Meanwhile, widowed patients with G-NEN had the highest risk of death compared with single, married, and divorced/separated patients.

## Background

Gastric neuroendocrine neoplasms (G-NENs) comprise a heterogeneous collective of tumours arising from the enterochromaffin-like cell, and account for approximately 7% of all neuroendocrine neoplasms [1]. In the past few decades, statisticians have witnessed a tenfold rise in the incidence of G-NEN, possibly due to progressed endoscopic screening skills and increased pathologic experience [2, 3]. G-NEN can be subdivided into three subtypes: type I associated with autoimmune atrophic gastritis, type II associated with Zolinger-Ellison syndrome/gastrinoma, and type III occurring sporadic without hypergastrinemia [4].

Nowadays, many clinicians and nurses mainly focused on clinicopathological characteristics, without taking the impact of psychological and social factors into consideration. In reality, these sociopsychological factors do have an influence on patient outcomes [5]. Marriage is one of the most important source of social support, which affects physical health through integrative physiological mechanisms [6]. Previous studies have pointed out that married patients tend to have better survival outcome in several cancer types [7–15]. However, whether marriage has a “protective” effect for G-NEN patients has not yet been established. In the present study, we examined the data from the Surveillance, Epidemiology, and End Results (SEER) cancer registry database to assess the effects of marital status on outcomes of patients with G-NEN.

## Methods

### Data sources and study population

The analysis was performed based on data obtained from the SEER registry. Using the National Cancer Institute’s SEER∗Stat software (Version 8.3.5), we identified G-NEN patients diagnosed from 1973 to 2015 with a known marital status. Primary site codes C16.0 to C16.9 and histological type codes were 8153/3: Gastrinoma, malignant, 8240/3: Carcinoid tumour, NOS, 8241/3: Enterochromaffin cell carcinoid, 8242/3: Enterochromaffin-like cell tumour, malignant, 8246/3: Neuroendocrine carcinoma, NOS, and 8249/3: Atypical carcinoid tumour, according to International Classification of Diseases for Oncology, Third Edition (ICD-O-3). The diagnosis of G-NENs was based on CS Schema v0204+ which classification as NETstomach. However, because of the data source and the study design, the classification into three clinical subtypes of G-NEN according to international guidelines [16] was not feasible in this study. Patients with nonprimary G-NET were excluded. The cause of death and survival of all patients were clearly known.

We have got permission to access the research data in SEER database and the reference number was 14,827-Nov2017. Since this was a retrospective cohort study, no ethical approval was required for analyses of these non-identifiable data.

### Statistical analysis

The clinical characteristics of the patients with G-NEN were presented with descriptive statistics. The categorical variable was presented with number (%). Chi-square tests were used to examine the association between marital status and other variables. Overall survival (OS), and cause-specific survival (CSS) rates were examined using the Kaplan-Meier method with log-rank tests.

Propensity scores (PSs) were estimated via a multivariable logistic regression model to balance 2 groups (married/unmarried) with respect to age at diagnosis, sex, year of diagnosis, ethnicity, tumour grade, and tumour stage. We then matched married and unmarried patients who had very similar PSs. 1:1 PS-matching was conducted using the nearest-neighbour algorithm with a caliper width of 0.01. Upon obtaining satisfactory subjects’ characteristics between married/unmarried groups, the hazard ratios (HRs) and 95% confidence intervals (CIs) of marital status over OS and CSS was estimated via a Cox proportional hazards regression model in all subjects and PS-matched cohort. The Kaplan-Meier survival curves were also plotted.

All statistical tests were 2-sided, and a value of P less than 0.05 was considered statistically significant. Statistical analyses were performed using the Statistical Product and Service Solutions (SPSS version 22.0; IBM Corporation, Armonk, NY, USA), and R (version 3.4.3; R Development Core Team, http://www.r-project.org).

## Results

### Patient characteristics

In total, 3947 patients with G-NEN who satisfied the inclusion criteria, comprising 2377 (60.2%) married patients and 1570 (39.8%) unmarried subjects, were identified in the SEER database. Of the unmarried subjects, 408 (10.3%) were divorced or separated, 646 (16.4%) were single, and 516 (13.1%) were widowed. Demographic and clinicopathological characteristics of these patients were described in Table 1, stratified by marital statuses. Chi-square tests showed significant differences in most variables, including age at diagnosis (*P* < 0.001), sex (*P* < 0.001), year at diagnosis (*P <* 0.001), ethnicity (*P* < 0.001), tumour size (*P* = 0.02), and surgery performed (*P* < 0.001).

**Table 1:** Characteristics of patients with gastric neuroendocrine tumour in SEER database before propensity score matching

Variable	Overall (***n*** = 3947)	Married (***n*** = 2377; 60.2%)	Divorced/Separated (***n*** = 408; 10.3%)	Single (***n*** = 646; 16.4%)	Widowed (***n*** = 516; 13.1%)	***P***
**Demographic parameters**						
Age at diagnosis						< 0.001
≤ 50	938	574 (61.2%)	90 (9.6%)	271 (28.9%)	3 (0.3%)	
51–70	1930	1243 (64.4%)	240 (12.4%)	293 (15.2%)	154 (8.0%)	
> 70	1079	560 (51.9%)	78 (7.2%)	82 (7.6%)	359 (33.3%)	
Sex						< 0.001
Male	1571	1121 (72.1%)	135 (8.2%)	248 (14.4%)	67 (5.3%)	
Female	2376	1256 (51.7%)	273 (12.2%)	398 (16.4%)	449 (19.8%)	
Year of diagnosis						< 0.001
1973–1995	254	172 (67.7%)	19 (7.5%)	26 (10.2%)	37 (14.6%)	
1996–2005	1120	659 (58.8%)	107 (9.6%)	166 (14.8%)	188 (16.8%)	
2006–2010	1127	686 (60.9%)	118 (10.5%)	184 (16.3%)	139 (12.3%)	
2011–2015	1446	860 (59.5%)	164 (11.3%)	270 (18.7%)	152 (10.5%)	
Ethnicity						< 0.001
White	3099	1945 (62.8%)	306 (9.9%)	456 (14.7%)	392 (12.6%)	
Black	563	234 (41.6%)	82 (14.6%)	158 (28.1%)	89 (15.8%)	
Others	252	175 (69.4%)	20 (7.9%)	26 (10.3%)	31 (12.3%)	
Unknown	33	23 (69.7%)	0 (0%)	6 (18.2%)	4 (12.1%)	
**Clinicopathological parameters**						
Grade						0.07
Well differentiated	1144	679 (59.4%)	125 (10.9%)	210 (18.4%)	130 (11.4%)	
Moderately differentiated	258	168 (65.1%)	24 (9.3%)	45 (17.4%)	21 (8.1%)	
Poorly differentiated	285	166 (58.2%)	33 (11.6%)	43 (15.1%)	43 (15.1%)	
Undifferentiated	73	42 (57.5%)	5 (6.8%)	13 (17.8%)	13 (17.8%)	
Unknown	2187	1322 (60.4%)	221 (10.1%)	335 (15.3%)	309 (14.1%)	
Tumour stage						0.19
Localized	2635	1612 (61.2%)	278 (10.6%)	416 (15.8%)	329 (12.5%)	
Regional	241	151 (62.7%)	27 (11.2%)	40 (16.6%)	23 (9.5%)	
Distant	463	271 (58.5%)	43 (9.3%)	81 (17.5%)	68 (14.7%)	
Unknown	608	343 (56.4%)	60 (9.9%)	109 (17.9%)	96 (15.8%)	
Size (cm)						0.02
≤ 5	79	57 (72.2%)	8 (10.1%)	5 (6.3%)	9 (11.4%)	
5.1–10.0	104	60 (57.7%)	7 (6.7%)	18 (17.3%)	19 (18.3%)	
> 10.0	229	146 (63.8%)	23 (10.0%)	22 (9.6%)	38 (16.6%)	
Unknown	3535	2114 (59.8%)	370 (10.5%)	601 (17.0%)	450 (12.7%)	
Surgery						< 0.001
Performed	2391	1498 (62.7%)	244 (10.2%)	376 (15.7%)	273 (11.4%)	
Not Performed	1482	841 (56.7%)	152 (10.3%)	252 (17.0%)	237 (16.0%)	
Unknown	74	38 (51.4%)	12 (16.2%)	18 (24.3%)	6 (8.1%)	

### The effects of marital status on overall and cause-specific survival

**Fig. 1:**
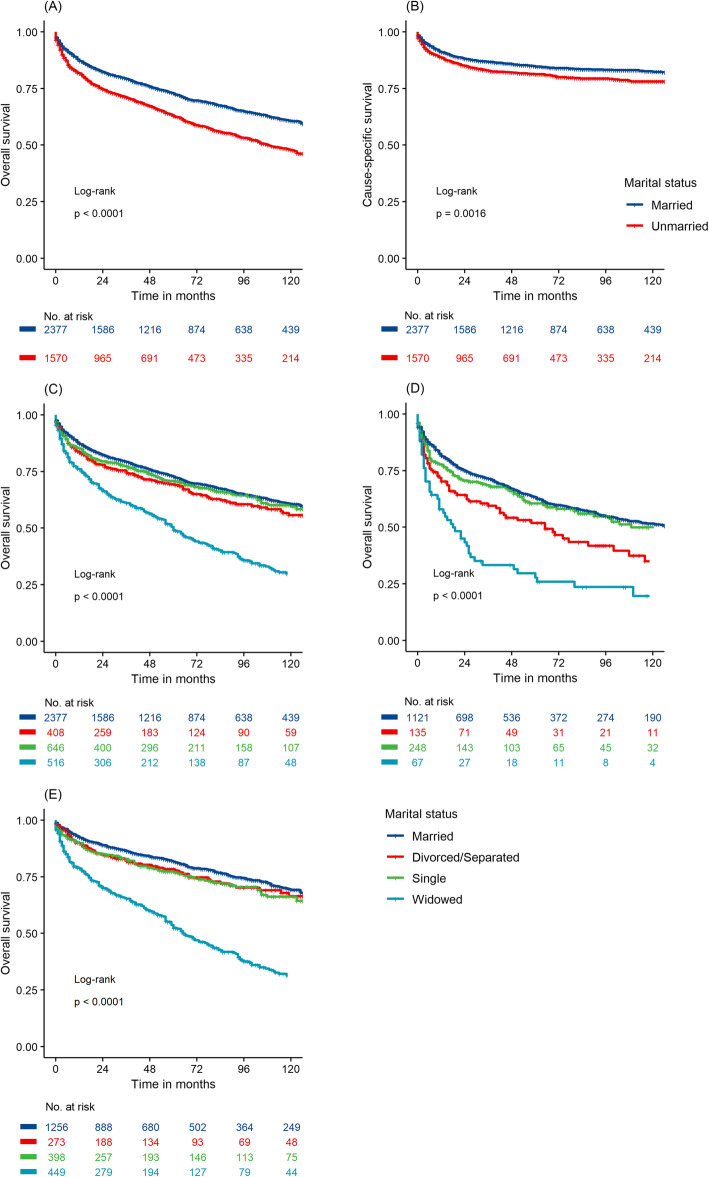
Kaplan-Meier survival curves of G-NEN patients according to marital status. **a**. overall survival between married and unmarried patients; **b** G-NEN cause specific survival between married and unmarried patients; **c**. overall survival among single, married, widowed, and divorced/seperated patients; **d** overall survival of male patients; **e**. overall survival of female patients

We applied Kaplan-Meier curves to evaluate the OS rates of G-NEN patients. As shown in Fig. 1a, unmarried status was associated with worse prognosis compared to married status according to the Cox regression model (HR 1.47, 95% CI 1.33–1.64, *P* < 0.001). After adjusting baseline parameters, including age, sex, year at diagnosis, race, tumour grade, tumour size, and surgery performed, unmarried patients still had poorer prognosis than married counterparts (HR 1.49, 95% CI 1.33–1.67, *P* < 0.001). The CSS rates of G-NEN patients were also displayed by plotting Kaplan-Meier curves. As shown in Fig. 1b, unmarried status contributed to unfavourable prognosis (HR 1.29, 95% CI 1.10–1.51, *P* = 0.002) according to the Cox model and even after adjusting confounding factors (HR 1.29, 95% CI 1.09–1.54, *P* = 0.003).

To explore whether different unmarried status led to worse prognosis than married status, we divided unmarried subjects into three subgroups: the divorced/separated, single and widowed. On univariable analysis, windowed patients had a statistically significant higher risk of all-cause mortality (HR 3.35, 95% CI 2.05–2.68, *P* < 0.001). As shown in Fig. 1c, compared with married patients, windows had significantly lower OS rate. On multivariable analysis, unmarried status (including single marital status) remained an independent prognostic factor for increased risk of all-cause mortality, while single status did not indicate higher risk of cancer-specific death compared to married G-NEN patients.

In addition, age, sex, tumour grade, tumour stage, and surgery performed were validated as independent prognosis factors for OS and CSS in the multivariate Cox analyses. The detailed description of each prognostic factor is displayed in Table 2. We also explored the association between marital status and survival only in patients well-differentiated tumours. As displayed in Table 3, both single (HR 1.55, 95% CI 1.03–2.32, *P* = 0.03) and widowed (HR 1.84, 95% CI 1.21–2.78, *P* = 0.004) patients were associated with decreased survival time, compared with married counterparts (P for trend = 0.02), after adjusting for known confounders.

**Table 2:** Cox proportional hazards model assessing factors associated with overall survival (OS) and cause-specific survival (CSS) before propensity score matching

Variable	OS				CSS			
	Crude HR (95% CI)	***P***	Adjusted HR (95% CI)	***P***	Crude HR (95% CI)	***P***	Adjusted HR (95% CI)	***P***
Marital status								
Married	1 [Reference]		1 [Reference]		1 [Reference]		1 [Reference]	
Divorced/Separated	1.19 (1.00–1.43)	0.06	1.44 (1.20–1.74)	< 0.001	1.09 (0.83–1.43)	0.56	1.35 (1.02–1.80)	0.04
Single	1.06 (0.91–1.24)	0.44	1.43 (1.22–1.68)	< 0.001	1.05 (0.83–1.31)	0.71	1.22 (0.96–1.56)	0.11
Widowed	3.35 (2.05–2.68)	< 0.001	1.56 (1.35–1.81)	< 0.001	1.80 (1.46–2.22)	< 0.001	1.33 (1.05–1.68)	0.02
Age at diagnosis								
≤ 50	1 [Reference]		1 [Reference]		1 [Reference]		1 [Reference]	
51–70	2.10 (1.76–2.50)	< 0.001	2.16 (1.80–2.58)	< 0.001	1.55 (1.22–1.96)	< 0.001	1.54 (1.20–1.96)	0.001
> 70	5.75 (4.83–6.84)	< 0.001	5.28 (4.38–6.38)	< 0.001	3.27 (2.57–4.15)	< 0.001	2.76 (2.12–3.61)	< 0.001
Sex								
Male	1.56 (1.41–1.73)	< 0.001	1.48 (1.32–1.65)	< 0.001	2.15 (1.84–2.52)	< 0.001	1.30 (1.09–1.55)	0.003
Female	1 [Reference]		1 [Reference]		1 [Reference]		1 [Reference]	
Year of diagnosis								
1973–1995	1.70 (1.38–2.10)	< 0.001	1.93 (1.54–2.42)	< 0.001	1.69 (1.25–2.29)	0.001	1.87 (1.34–2.60)	< 0.001
1996–2005	1.58 (1.35–1.86)	< 0.001	1.66 (1.40–1.98)	< 0.001	1.46 (1.17–1.81)	0.001	1.56 (1.22–2.00)	< 0.001
2006–2010	1.14 (0.96–1.34)	0.15	1.15 (0.97–1.36)	0.12	1.16 (0.93–1.46)	0.19	1.17 (0.93–1.47)	0.19
2011–2015	1 [Reference]		1 [Reference]		1 [Reference]		1 [Reference]	
Ethnicity								
White	1 [Reference]		1 [Reference]		1 [Reference]		1 [Reference]	
Black	1.12 (0.97–1.30)	0.11	1.18 (1.02–1.37)	0.03	1.14 (0.91–1.42)	0.25	1.18 (0.89–1.40)	0.34
Others	1.05 (0.85–1.30)	0.64	0.97 (0.78–1.20)	0.78	1.63 (1.24–2.14)	< 0.001	1.23 (0.93–1.63)	0.15
Grade								
Well differentiated	1 [Reference]		1 [Reference]		1 [Reference]		1 [Reference]	
Moderately differentiated	1.76 (1.33–2.34)	< 0.001	1.60 (1.20–2.12)	< 0.001	2.99 (1.95–4.57)	< 0.001	2.05 (1.33–3.15)	0.001
Poorly differentiated	8.46 (6.98–10.25)	< 0.001	2.93 (2.38–3.62)	< 0.001	23.87 (17.80–32.02)	< 0.001	4.40 (3.21–6.02)	< 0.001
Undifferentiated	8.11 (6.00–10.96)	< 0.001	3.51 (2.56–4.80)	< 0.001	22.06 (15.04–32.36)	< 0.001	4.71 (3.16–7.02)	< 0.001
Unknown	1.51 (1.28–1.77)	< 0.001	1.11 (0.94–1.31)	0.23	1.84 (1.38–2.45)	< 0.001	1.30 (0.96–1.77)	0.09
Tumour stage								
Localized	1 [Reference]		1 [Reference]		1 [Reference]		1 [Reference]	
Regional	2.83 (2.35–3.41)	< 0.001	2.16 (1.77–2.64)	< 0.001	11.12 (8.54–14.47)	< 0.001	7.15 (5.37–9.52)	< 0.001
Distant	7.54 (6.62–8.58)	< 0.001	4.36 (3.73–5.09)	< 0.001	32.34 (26.20–39.92)	< 0.001	15.83 (12.37–20.27)	< 0.001
Unknown	1.37 (1.17–1.60)	< 0.001	0.93 (0.79–1.10)	0.42	1.97 (1.44–2.71)	< 0.001	1.35 (0.97–1.88)	0.08
Size (cm)								
≤ 5	1 [Reference]		1 [Reference]		1 [Reference]		1 [Reference]	
5.1–10.0	0.90 (0.60–1.35)	0.62	0.97 (0.64–1.45)	0.87	1.53 (0.62–3.79)	0.36	1.62 (0.65–4.02)	0.30
> 10.0	1.71 (1.22–2.39)	0.002	0.94 (0.67–1.33)	0.74	6.37 (2.96–13.70)	< 0.001	1.58 (0.73–3.45)	0.25
Unknown	1.10 (0.81–1.49)	0.55	0.96 (0.70–1.32)	0.81	2.26 (1.07–4.78)	0.03	1.17 (0.55–2.50)	0.69
Surgery								
Performed	1 [Reference]		1 [Reference]		1 [Reference]		1 [Reference]	
Not Performed	2.22 (1.99–2.46)	< 0.001	1.67 (1.47–1.89)	< 0.001	3.13 (2.65–3.69)	< 0.001	1.95 (1.59–2.39)	< 0.001
Unknown	1.71 (1.15–2.56)	0.008	2.03 (1.36–3.02)	0.001	2.22 (1.27–3.88)	0.005	2.42 (1.36–4.29)	0.003

**Table 3:** Cox proportional hazards model assessing association of marital status with overall survival in well-differentiated tumors

	Crude HR (95% CI)	***P***	Adjusted HR (95% CI)	***P***
Marital status		< 0.001^*^		0.02^*^
Married	1 [Reference]		1 [Reference]	
Divorced/Separated	1.15 (0.70–1.88)	0.58	1.29 (0.78–2.13)	0.33
Single	1.32 (0.90–1.95)	0.16	1.55 (1.03–2.32)	0.03
Widowed	2.56 (1.78–3.67)	< 0.001	1.84 (1.21–2.78)	0.004

*HR* hazard ratio, *CI* confidence interval. Adjusted HRs were calculated after adjustments for age, sex, race, tumor stage, tumor size, and surgery status

^*^P for trend

### Subgroup analysis of the effect of marital status stratified by gender

Since widowed patients had the poorest OS, we analysed whether unmarried status, especially widowed status contributed to the poor survival rates in the subgroups of G-NEN patients stratified by gender. As shown in Table 4, marital status was found to be an independent prognostic factor of OS in both male and female G-NEN patients according to the log-rank tests and Cox regression analysis (Fig. 1d, e). Particularly, widowhood affected the prognosis more in women than in men.

**Table 4:** Univariate and multivariate survival analysis for marital status on overall survival in male and female G-NET patients before propensity score matching

Gender	5-year OS	Univariate analysis		Multivariate analysis	
		Log-rank χ^**2**^ test	***P***	Adjusted HR (95% CI)	***P***
Male		50.3	< 0.001		
Married	67.9%			1 [Reference]	
Divorced/Separated	57.0%			1.95 (1.50–2.52)	< 0.001
Single	66.1%			1.31 (1.04–1.64)	0.02
Widowed	31.3%			1.36 (1.01–1.84)	0.04
Female		142.9	< 0.001		
Married	85.0%			1 [Reference]	
Divorced/Separated	81.7%			1.13 (0.86–1.47)	0.38
Single	81.2%			1.51 (1.20–1.91)	0.001
Widowed	58.35%			1.58 (1.31–1.90)	< 0.001

### Clinical outcomes after propensity score matching

To further confirm the findings that married G-NEN patients survived longer and to minimize bias in the previous analysis, we conducted a PS-matching analysis. Using a 1:1 PS-matching method, we matched 506 unmarried patients with 506 married patients. As shown in Table 5, all the baseline variables were clearly well matched (all *P* > 0.05).

**Table 5:** Characteristics of patients with gastric neuroendocrine tumour in SEER database after propensity score matching.

Variable	Overall (***n*** = 1012)	Married (***n*** = 506)	Unmarried (***n*** = 506)	***P***
Age at diagnosis				0.64
≤ 50	241	126 (52.3%)	115 (47.7%)	
51–70	515	257 (49.9%)	258 (50.1%)	
> 70	256	123 (48.0%)	133 (52.0%)	
Sex				0.95
Male	373	187 (50.1%)	186 (49.9%)	
Female	639	319 (49.9%)	320 (50.1%)	
Year of diagnosis				0.62
1973–1995	11	5 (45.5%)	6 (54.5%)	
1996–2005	141	77 (54.6%)	64 (45.4%)	
2006–2010	254	129 (50.8%)	125 (49.2%)	
2011–2015	606	295 (48.7%)	311 (51.3%)	
Ethnicity				0.21
White	801	395 (49.3%)	406 (50.7%)	
Black	156	87 (55.8%)	69 (44.2%)	
Others	55	24 (43.6%)	31 (56.4%)	
Grade				0.20
Well differentiated	719	341 (47.4%)	378 (52.6%)	
Moderately differentiated	142	76 (53.5%)	66 (46.5%)	
Poorly differentiated	151	89 (58.9%)	62 (41.1%)	
Tumor stage				0.10
Localized	801	390 (48.7%)	411 (51.3%)	
Regional	71	34 (47.9%)	37 (52.1%)	
Distant	140	82 (58.6%)	58 (41.4%)	

**Fig. 2:**
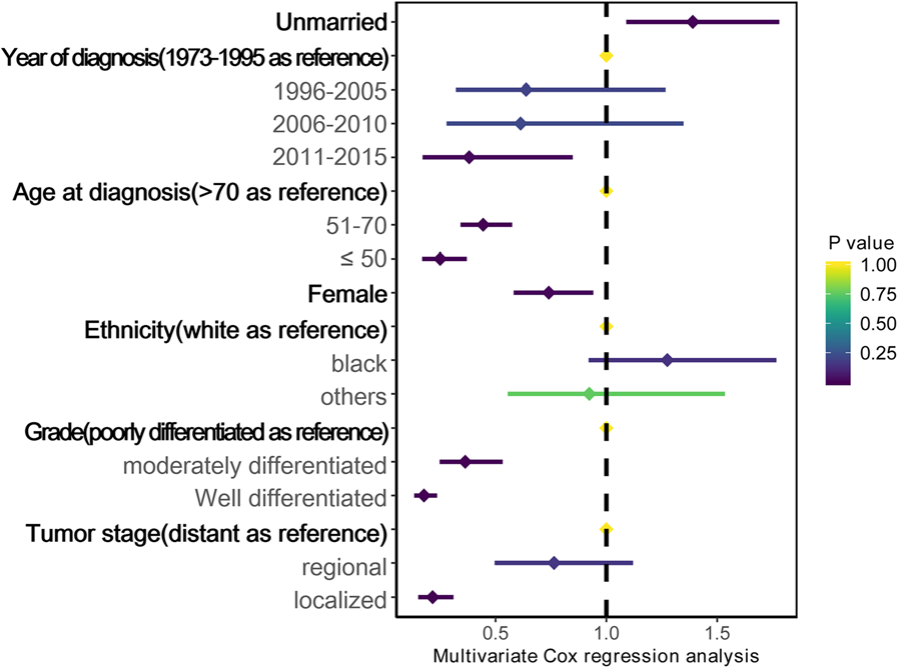
Forest plot presenting the contribution of unmarried status compared with that of married status to the overall survival rates of patients in the PS-matched cohort. HR > 1 with *P* < 0.05 meant that unmarried status contributed significantly to poorer survival than married status

**Fig. 3:**
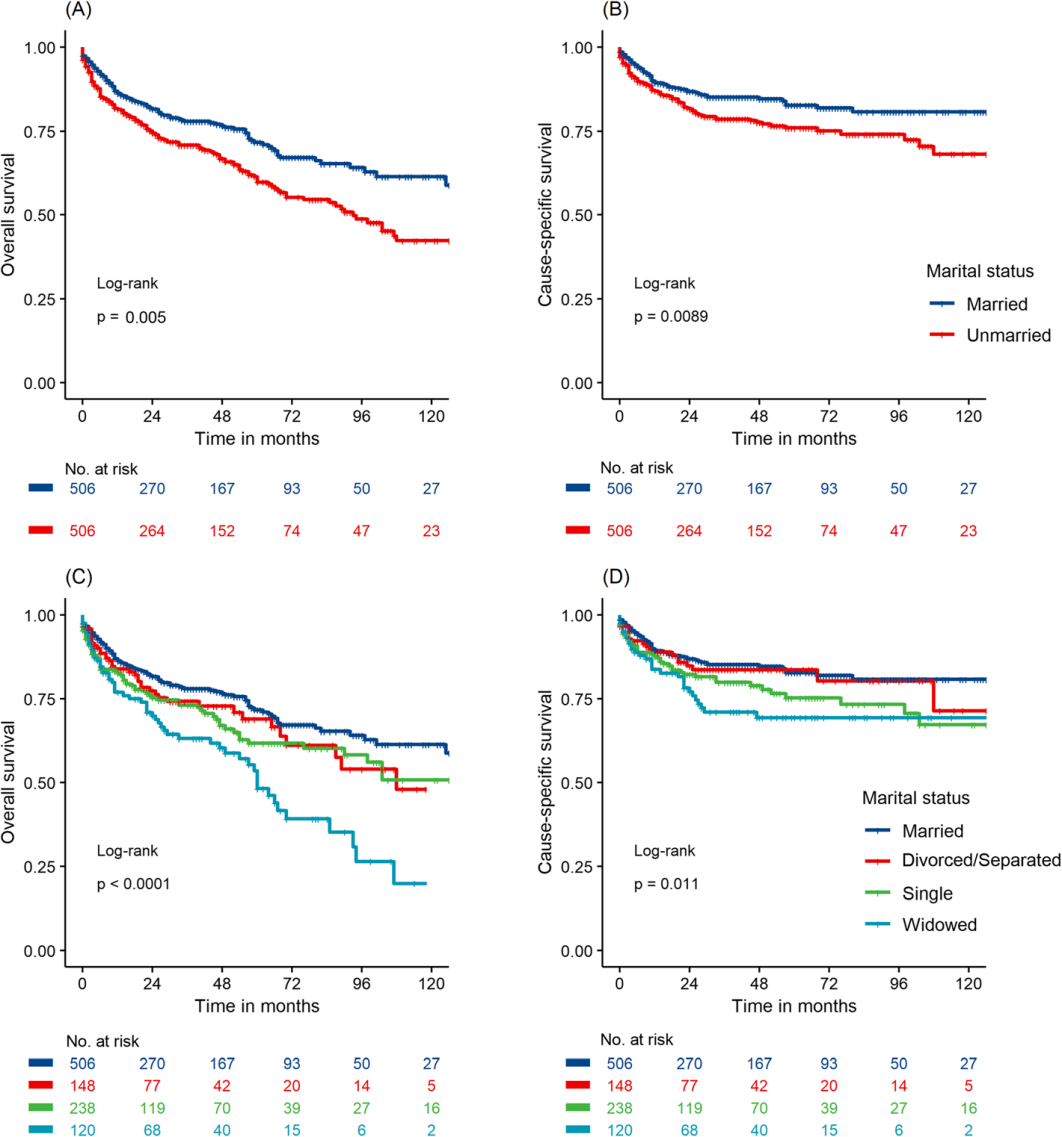
Kaplan-Meier survival curves of G-NEN patients according to marital status after propensity score matching. **a**. overall survival between married and unmarried patients; **b** G-NEN cause specific survival between married and unmarried patients; **c**. overall survival among single, married, widowed, and divorced/seperated patients; **d** G-NEN cause specific survival among single, married, widowed, and divorced/seperated patients

Although the HR was not higher after matching the data than before, unmarried patients still shown poorer OS (HR 1.51, 95% CI 1.19–1.90, *P* = 0.001) and CSS (HR 1.50, 95% CI 1.10–2.05, *P* = 0.01) in univariate Cox model. In multivariate analysis (Fig. 2), unmarried status was still linked with significantly worse OS (HR 1.39, 95% CI 1.09–1.78, *P* = 0.008). As shown in Fig. 3a and b, survival curves for OS and CSS indicated that married patients showed significantly better survival than their unmarried counterparts. Compared with married patients, widowed patients had a significant reduction in both OS and CSS rate (Fig. 3c, d).

## Discussion

In this study, we assessed the impact of marital status at diagnosis on survival outcomes in a SEER cohort of G-NEN patients. Based on relatively large sample size and PS-matched dataset, our study provided results with high validity and reliability. Being married was indicated to exert a protective effect on survival compared to any unmarried status.

The diagnosis of cancer exposes an individual to chronic psychosocial stress, which triggers fight-or-flight responses by activating the hypothalamic-pituitary-adrenal axis. From a physiology perspective, psychological stress increases epinephrine, prostaglandins, and glucocorticoid levels, and reduces NK cells and cytotoxic T cells activity [17–19]. Then stress induces immune suppression, contributing to tumour proliferation, progression, and metastasis [20, 21]. A cell line study of ovarian cancer demonstrated that stress hormones can also enhance the capacity of tumour cells to invade the extracellular matrix, contributing to tumour metastasis [22]. The detailed mechanism for protective role of marriage on neuroendocrine tumours might be explored in further experimental studies. Typically, oncological patients deny, feel angry, bargain, experience depression, and then gradually accept the reality. Social support, or supportive social network, is greatly needed throughout this process. With emotional support of their spouse, married patients experience less stress and despair [23]. Additionally, patients with less psychosocial stress have better compliance to medical recommendations [24]. Spousal encouragement may increase G-NEN patients’ willingness to survive, and they are more likely to receive treatments like surgery and/or chemotherapy.

With the transformation from biomedical model to biopsychosocial model of illness [25], the importance of sociopsychological factors on oncological diseases has gained increasing attention. Positive psychosocial factors can alleviate the pain and worries of cancer patients, thus improving the treatment compliance, treatment effect, quality of life and survival rate. Therefore, it is of great significance to fully understand the relationship between prognosis of tumour patients and psychosocial factors and to monitor the psychological changes of tumour patients. Sociopsychological factors, including marital status, can impact tumour development and survival of oncological diseases through the regulation of endocrine and immune systems. Our results show that all unmarried groups showed poorer survival outcome compared with the married group, but windowed G-NEN patients have the poorest prognosis, which is also demonstrated in studies regarding gastrointestinal stromal tumour, gastric cancer, nasopharyngeal carcinoma, and rectal cancer [26–29]. Single and separated G-NEN patients tend to be more prepared to build social support networks other than marriage compared to widowed patients. As such, clinicians, nurses, and health care workers need to pay more attention to widowed patients’ emotional need, communicate more with the widowed, and provide them with necessary social support in clinical practice.

Despite of the strengths of this study including large sample size, subgroup analysis, and PS-matching method, there were some potential limitations. First, we ignored effect of the quality of marital life among G-NEN patients in the analysis. This may cause bias since unsatisfactory marriage can result in immune dysregulation [30]. Previous study revealed that marital relationship may change after cancer diagnosis [31]. The SEER database did not provide information on change of marital status after G-NEN diagnosis. Another notable drawback was the inability to classify patients according with the clinical subgroups (Type I, II, III) according with international guidelines [16], where type III tumour showed markedly worse outcome than others. Since type I and II of G-NENs comprise vast majority of well-differentiated tumours in general, and most type III tumour can be classified into poorly-differentiated neuroendocrine carcinoma, we tried to compensate for this limitation by validating the prognostic effect of marital status in well-differentiated tumours only. Besides, we failed to adjust some recognized prognostic parameters such as chemotherapy and radiation in the regression model due to lack of detailed information in the database.

In addition to marital status, many other socioeconomic factors (e.g. household income and medical insurance status) and sociopsychological factors may also play a role in G-NEN patients’ outcomes, which warrant further investigation. Moreover, further in-depth investigations according with different G-NEN types are needed to better understand the meaning of the findings in the present study.

## Conclusions

In summary, our study found that marital status was an independent prognostic factor among G-NEN patients, and married individuals enjoyed significant survival benefits than those unmarried. Particularly, widowed G-NEN patients suffer the highest mortality risk. It is necessary to provide timely psychological intervention and social support for unmarried, especially widowed G-NEN patients in clinical practice. However, our results should be interpreted with caution since the inability to classify patients into the three clinical types of G-NEN in this study.

## Data Availability

The data used to support the findings of this study are available from the SEER database (seer.cancer.gov).

## Abbreviations

G-NEN: Gastric neuroendocrine neoplasms
SEER: Surveillance, Epidemiology, and End Results
ICD-O-3: International Classification of Diseases for Oncology, Third Edition
OS: Overall survival
CSS: Cause-specific survival
HR: Hazard ratios
CI: Confidence interval
